# Pacing for the Suppression of Paroxysmal Atrial Fibrillation in an 87-year-old Patient

**Published:** 2003-04-01

**Authors:** Paul A Levine, Robin Wachsner, Adel El-Bialy

**Affiliations:** Section of Cardiology, Olive View Medical Center, 14445 Olive View Drive, Sylmar CA 91342

## Abstract

**Background:**

Sinus node dysfunction, atrioventricular (AV) block and atrial fibrillation (AF) are associated with advanced age. Required therapy commonly includes pacemaker implantation.

**Methods:**

We report the course of therapy for an 87-year-old with symptomatic sinus node dysfunction and paroxysmal atrial fibrillation who was intolerant of drug therapy.

**Results:**

The patient received a pacemaker for treatment of sick sinus syndrome. She continued to have symptomatic episodes of AF and was intolerant of pharmacologic therapy despite adequate rate support provided by the pacemaker. The AF suppression algorithm in the pacemaker was enabled, resulting in the elimination all AF episodes effectively eliminating the need for antiarrhythmic medication. If this continues to stabilize her atrium, withdrawal of anticoagulation therapy is anticipated.

**Conclusions:**

The clinical presentation of sinus node dysfunction and related conduction abnormalities is common in the elderly. Pharmacologic management is often a challenge in the presence of the advanced age and concomitant disease processes. In individuals who have paroxysmal atrial fibrillation or are likely to develop this and who need a pacemaker for standard indications, the availability of an AF Suppression™ algorithm may facilitate their management without needed to use medications or being able to utilize lower doses of those medications.

## Clinical History

The patient is a frail 87-year-old woman with symptomatic sinus node dysfunction and documented paroxysmal atrial fibrillation (PAF). She presented with PAF and presyncopal spells. The presyncopal episodes coincided with episodes of sinus arrest occurring upon spontaneous termination of AF. Various antiarrhythmic agents (AA) were utilized including digoxin 0.125 mg/d with serum digoxin level of 1.0 ng/ml, atenolol starting at 25 mg QD, titrating up to 50 mg BID and sotalol 80 MG BID. All of these AA were either ineffective, exacerbated her bradyarrhythmia or caused intolerable side effects. She was also placed on coumadin 2.5 mg alternating with 5.0 mg due to the frequent episodes of AF, with a target INR of 2-3.

A pacemaker (St. Jude Medical Integrity™ μ DR) was implanted (October 21, 2001) to provide dual-chamber pacing (DDD mode) to manage her symptomatic bradycardic episodes and allow for the safe administration of pharmacologic therapy. The automatic mode switch function (AMS) was enabled to prevent tracking of the high atrial rate by the pacemaker. It also served as a diagnostic marker for recurrent and possibly clinically asymptomatic episodes of PAF. This function allows internal pacemaker diagnostic records to detail pacing and AMS events, enabling their quantification at patient follow-up.

 At five weeks, the pacing system was functioning appropriately, but the patient complained of fatigue. It was rationalized that the fatigue might be an inherent effect of her medications.

At this time, all medications except atenolol were discontinued and the atenolol was reduced to 25 mg QD, to facilitate control of the ventricular response during AF. Coumadin was continued.

At her follow-up visit 4 months post-implant, the patient reported a marked decrease in the frequency, but not total elimination of her palpitations. There was total elimination of her near syncopal spells. Standard DDD pacing appeared to have been effective in treating her presyncopal symptoms but at standard settings, episodes of paroxysmal atrial fibrillation continued. The AMS event log recorded 43 episodes, most being triggered by atrial rates > 300 bpm and consistent with AF. Although the cumulative episodes of atrial fibrillation accounted for < 1% of the rhythm activity since her last evaluation, she continued to complain of the palpitations. As she was intolerant of most medications, the AF Suppression™ algorithm integral to her implanted pacing system was enabled and a follow-up visit was scheduled in three months.

On her return, she reported feeling well with resolution of both palpitations fatigue. The AMS event log data showed no AMS episodes. It was opted to withdraw the low dose of atenolol at this time but continue the coumadin. Future plans include a follow up in six months and if AF remains suppressed, to discontinue coumadin anticoagulation.

## Discussion

The AF Suppression™ algorithm is a newly approved algorithm designed to provide a high percentage of atrial overdrive pacing which usurps the control of the atrial rate, either from the sinus node or an ectopic foci. The objective is reduced temporal dispersion of the atrial refractory period combined with overdrive suppression of ectopic beats, a common trigger for atrial tachyarrhythmias. AMS remains functional with the AF Suppression™ algorithm on, thus if AF occurs, AMS is activated facilitating management as this provides information as to the frequency and duration of each AMS episode. Based on the detected atrial rate, one can infer the mechanism of the atrial arrhythmia with the faster episodes being atrial fibrillation.

Sinus node dysfunction, AV block and AF progressively increase in frequency with advancing age [1]. Standard treatment for symptomatic bradyarrhythmias is pacing while rapid heart rhythms are commonly treated pharmacologically [2,3]. However, pharmacologic therapy is often a challenge in the very elderly ( > 85) due to alterations in pharmacokinetics and pharmacodynamics. Management of these patients becomes a complex task, particularly when the number of medications prescribed increases and one has to be concerned with adverse drug interactions and side effects [4]. 

Device therapy is the standard approach to the management of a symptomatic bradycardia. The majority of pacemaker patients are elderly, with more than 85% of pacemaker recipients being at least 64 years old [5]. Thus, as the technology and features of pacemakers improve, it is important for the clinician to understand how these technological advances can optimize patient care.

AF is specific arrhythmia associated with age, either by itself or in conjunction with other conduction abnormalities. The prevalence of AF doubles for each decade of life after age 60 [6]. Although not lethal, patients with AF have twice the risk for death, and if not on anticoagulant therapy, a five-fold risk for stroke. This risk escalates in patients 75 and older [7]. Quality of life is also compromised as a consequence of the loss of atrial transport and the irregular and often rapid ventricular rates. The initial treatment of AF is pharmacologic designed to either restore sinus rhythm or to control the ventricular response to this rhythm. In those individuals who have the bradycardia-tachycardia syndrome, dizziness and/or syncope are commonly associated with the protracted asystolic pauses following spontaneous termination of a paroxysmal atrial fibrillation episode. Pharmacologic therapy may exacerbate these symptoms by further lengthening the sinus node recovery time following spontaneous termination of an episode as well as exacerbating the underlying bradycardia when in sinus rhythm. A common treatment for PAF associated with bradycardia-tachycardia syndrome is implantation of a permanent pacemaker in combination with drugs, as was done in this case [8]. Although drug therapy is the initial therapy, the majority of cardiovascular agents, can have deleterious effects in the geriatric population requiring careful attention to management [9].

The decision at the time of implantation was to select a device with a commercially available overdrive suppression algorithm although there was no way to determine whether or not it would be successful in this patient prior to its implantation. The addition of the commercially approved AF Suppression™ algorithm proved to be a valuable adjunct to this patient's management allowing us to discontinue a multiplicity of medications. We cannot determine whether it was stabilization of her atrial rhythm or elimination of her polypharmacy that accounted for her clinical improvement but both were made possible by technologic advances in the form of the AF Suppression algorithm.

While the algorithm may not be 100% effective in all patients, any reduction in the number of AF episodes is beneficial. In this case, standard pacing resulted in a marked improvement for the patient, but episodes of symptomatic and asymptomatic PAF continued. The ability to then enable the AF Suppression™ algorithm provided additional benefit, further improving this patient's quality of life as well as allowing discontinuation of her current antiarrhythmic medications. Although this is an isolated case, the use of a pacemaker containing the AF Suppression™ algorithm should be considered in all patients undergoing device implant for a symptomatic bradycardia, particularly when there is a prior history of atrial fibrillation or they are at increased risk of atrial fibrillation which is more likely when the indication for pacing is sinus node dysfunction.
